# Advance at a glance: contributions to cardio-oncology

**DOI:** 10.1093/ehjopen/oeaf075

**Published:** 2025-06-20

**Authors:** Joerg Herrmann

**Affiliations:** Department of Cardiovascular Medicine, Mayo Clinic, 200 First Street SW, Rochester, MN 55905, USA

**This editorial refers to the European Heart Journal Open Article Collection “Advances in Cardio-Oncology”, available at: https://academic.oup.com/ehjopen/pages/advances-in-cardioncology.**

Cardiovascular diseases (CVD) and cancer are the leading causes of death worldwide, responsible, together with other non-communicable diseases (NCDs), for over three-quarters of deaths in individuals age 30 to 70 in the world in 2019. This burden is expected to rise over the next decades as communicable diseases are progressively controlled and risk factors for NCDs are on the rise, in particular aging.^1^ Over 20 years ago, governments adopted the UN Political Declaration on NCDs and committed to action to combat premature mortality from NCDs. The 2030 Sustainable Development Goal is to reduce the probability of dying between ages 30 and 70 from NCDs by one-third by 2030. Over the 20-year period, indeed, CVD- and cancer-related mortalities have declined, by 30–60% and 20–30%, respectively, in high income countries, but less so and less consistently across middle- and low-income countries. CVD account for twice as many death as cancer, and by 2050, it is estimated that there will be as many deaths due to CVDs as there will be new cases of cancer worldwide: 35 million.^2,3^

These numbers are a testimony to the powerful changes in development across the globe and the efforts of modern medicine from screening to prevention and treatment. These trends though, unmistakeably, also leave us with the need to prepare to care for patients with several diseases over long periods of time, especially cancer and CVD, and the management of either can impact the outcome of the other and overall. To raise awareness for this interplay, EHJ Open, in August 2024, launched a collection of articles on what has become known as cardio-oncology. These articles illustrate trends of CVD incidence and mortality in specific cancer populations, effectiveness of beta-blocker, angiotensin converting enzyme (ACE) inhibitors, or angiotensin receptor blockers (ARBs) for the prevention of cardiomyopathy (CM) and heart failure in cancer survivors, how ST segment elevation myocardial infarction (STEMI) patients with cancer differ from those without cancer, and how scores commonly used in the management of patients with atrial fibrillation (AF) may or may not apply to those with vs. those without malignancy.

In the first paper to highlight, Engebretsen *et al.*^4^ defined the prevalence and incidence of eight CVD diagnoses among Norwegian female breast cancer patients diagnosed between 2013 and 2020, compared with a nationwide control cohort of women without breast cancer.^4^ Women with breast cancer had a higher risk of pulmonary embolism (three-fold), AF (1.5-fold), other cardiac arrhythmias (1.4-fold), as well as heart failure (two-fold), hypertensive heart disease (1.8-fold), and heart valve disease (two-fold) over time after their cancer diagnosis. They did not have a higher incidence of coronary and peripheral vascular disease, however. Worthwhile to point out further is the fact that even for CVDs with notable differences, incidence rates were low: 0.3% of patients developed HF, 2% PE; AF and hypertensive heart disease with 3.8% and 10.3% were the most common entities. Just over 60% of breast cancer patients died of cancer. These data are important in illustrating the higher burden of many but not all CVDs in breast cancer patients. Hypertensive heart disease, in particular, is very common in breast cancer women and needs attention by primary care, oncology, and cardiology providers. Hypertension remains the single most important modifiable risk factor for cardiotoxicity across the entire continuum of care for the cancer patient.

Using the Centers for Disease Control and Prevention’s (CDC’s) Wide-Ranging Online Data for Epidemiologic Research database, Agarwal *et al.*^5^ analyzed the trends in CVD–related mortality in patients with myeloproliferative neoplasms (MPNs) from 1999 to 2020.^5^ Importantly, they noticed a decreasing trend in CVD-related age-adjusted mortality rates (AAMRs) over the years. Men had a consistently higher AAMR than women, and Non-Hispanics (NHs) had the highest overall AAMR, followed by NH Blacks. These data are important because they may be a testimony to the importance of a multi-disciplinary approach to the care of these patients and may exemplify the impact cardio-oncology care can have for patients with these and other types of malignancies. There are not many studies which have attempted to quantify the significance of cardio-oncology services on patient outcomes. One from Thailand in breast cancer patients undergoing anthracycline-based therapy is to be highlighted though, which demonstrated a reduction in cardiovascular disease and mortality burden following the introduction of a cardio-oncology program.^6^

In another nationally linked cohort study of STEMI patients between January 2005 and March 2019, Dafaalla *et al.*^7^ evaluated the 1-year risk of major bleeding (any bleeding event requiring hospital admission) and re-infarction (according to the fourth Universal Definition of Myocardial Infarction).^7^ The incidence of major bleeding and re-infarction was higher in cancer patients at 1 year (6.5% vs. 3.5%, *P* < 0.001 and 5.7% vs. 5.1%, *P* = 0.01, respectively). The risk for both of these events peaked on the first days post-discharge and tapered gradually thereafter until reaching a plateau after 60 days. Major bleeding was particularly dominant in colon and prostate cancer, whereas re-infarction dominated in haematological malignancies. After adjusting for patients’ characteristics and comorbidities, cancer remained an independent risk factor for bleeding, but not for re-infarction. Major bleeding and re-infarction were both associated with a significant increase in all-cause death, and the prognostic impact of major bleeding (but not re-infarction) was higher in patients with than without active cancer. This study confirms and adds to prior studies on this topic.^8^ Beyond the recognition of CV death as the leading cause of death in the first 30 days and increased risk of HF admissions in the year to follow, which the authors reported on before, this study draws attention particularly to the increased incidence and implication of major bleeding in cancer patients with STEMI, particularly in the first 60 days after the event.^9,10^ It is a call to deliver the most optimal quality of care for these patients, which has not (always) been the case, as reflected in a lower metric of quality of care indicators in STEMI patients with cancer.^7,11^

Bleeding is also of particular concern for cancer patients with AF in consideration of anticoagulation. Scores have been used to define the risk-benefit constellation, namely the CHA2DS2-VASc score and the HAS-BLED score. Ajabnoor *et al.*^12^ evaluated the performance of these scores, their ability to predict ischaemic stroke and major bleeding events, in patients with AF and five types of cancer using a population-based electronic health record dataset linking primary and secondary care data and mortality records in England years 2009 through 2019.^12^ Overall, the CHA2DS2-VASc score performed similarly well (good to modest discrimination) in cancer patients (AUC 0.69–0.74, worst in lung, best in prostate cancer) than it did in non-cancer patients (AUC 0.73). The HAS-BLED score showed poor discriminatory ability in both non-cancer patients (AUC 0.61) and cancer patients (AUC 0.56–0.59, worst in breast, best in lung cancer and haematological malignancies). These are very important data for every practitioner bringing to attention the truly only modest performance of one of the most frequently used risk scores in medicine, the CHA2DS2-VASc score, as well as its companion, the HAS-BLED score. In fact, the latter was just a little better than a flip of a coin to predict bleeding in cancer patients in this study. This study is in agreement with the challenges reported with the use of these risk score in cancer patients.^13–15^ These patients remain at high risk of events, including both thrombotic and bleeding events, and their management will continue to require open discussions with the patient and shared decision making. The 2022 ESC cardio-oncology guidelines support the use of the CHA2DS2-VASc score but see the HAS-BLED score as but one element in the bleeding risk assessment, and the current study would support this approach.^16^

Last but not least, *Hindocha et al.*^17^ investigated the association between prescriptions for beta-blockers, ACE inhibitors, or ARBs in the year following a diagnosis of breast cancer or Non-Hodgkin’s lymphoma and subsequent risk of heart failure/cardiomyopathy (HF/CM) using primary care data collected from the Clinical Practice Research Datalink (CPRD GOLD) in the United Kingdom.^17^ In this population, the prescription of ARBs, ACEIs, and/or beta- blockers was not associated with the incidence of HF/CM, and stratified analyses provided no statistical evidence that the association between the prescription of ARBs, ACEIs, and/or beta-blockers and HF/CM incidence differed by cancer site, age at cancer diagnosis, radiotherapy, prior CVD disease, or years since cancer diagnosis. The crude incidence of HF/CM was approximately three times higher for individuals prescribed a beta-blocker, ARB, and/or ACEI compared with those who were not, but after adjusting for measured confounders, the strength of association was close to zero. Most certainly though, the use of these agents was not associated with a reduction in HF/CM. This observation challenges the common and historically grounded practice of using of these agents for cardio-protection in patients. One must point out though that the prescription of these medications very likely did not reflect dedicated efforts of prevention of cardiotoxicity but rather routine incidental use around the time of cancer treatment. This being said, other retrospective cohort studies have used a similar approach and noted beneficial effects.^18–20^ Prospective randomized clinical trials studies have yielded conflicting results, though aggregate, systematic reviews and meta-analyses over the past 10 years tend to support a benefit for beta-blocker and ACE inhibitors/ARBs as well as statins for cardioprotection.^21,22^

Defining and refining the optimal cardiovascular care of the cancer patient is a need today and will be even more so tomorrow and not only in Western countries but worldwide as developing countries see the transition to NCDs and developed countries to an aging society. Both, CVD present before and newly developing during cancer therapy, negatively impacts the survival prognosis, with patients experiencing both being most profoundly affected.^23^ Besides defining what to deliver, it is also important to define how to deliver the most optimal cardiovascular care for the cancer patient. The introduction of cardiology oncology services has been shown to improve outcomes, underscoring that knowledge of and dedication to this topic can make a difference.^6^ This collection of articles in EHJ Open is intended to contribute to these efforts, an advance at a glance.

## Data Availability

There are no new data associated with this article.
